# Altered PLCβ/IP_3_/Ca^2+^ Signaling Pathway Activated by GPRCs in Olfactory Neuronal Precursor Cells Derived from Patients Diagnosed with Schizophrenia

**DOI:** 10.3390/biomedicines12102343

**Published:** 2024-10-15

**Authors:** Zuly A. Sánchez-Florentino, Bianca S. Romero-Martínez, Edgar Flores-Soto, Luis M. Montaño, Bettina Sommer, Marcela Valdés-Tovar, Jesús Argueta, Eduardo Calixto, Arnoldo Aquino-Gálvez, Manuel Castillejos-López, Héctor Serrano, Juan C. Gomez-Verjan, Germán O. López-Riquelme, Gloria A. Benítez-King, Ruth Jaimez, Héctor Solís-Chagoyán

**Affiliations:** 1 Posgrado en Biología Experimental, Universidad Autónoma Metropolitana-Iztapalapa, Mexico City 09340, CP, Mexico; zulyarmandosf@gmail.com; 2 Laboratorio de Neurofarmacología, Subdirección de Investigaciones Clínicas, Instituto Nacional de Psiquiatría Ramón de la Fuente Muñiz, Mexico City 14370, CP, Mexico; jadclear@gmail.com (J.A.); bekin@imp.edu.mx (G.A.B.-K.); 3 Departamento de Farmacología, Facultad de Medicina, Universidad Nacional Autónoma de México, Mexico City 04510, CP, Mexico; biancasromero_@hotmail.com (B.S.R.-M.); edgarfloressoto@yahoo.com.mx (E.F.-S.); lmmr@unam.mx (L.M.M.); 4 Departamento de Investigación en Hiperreactividad Bronquial, Instituto Nacional de Enfermedades Respiratorias “Ismael Cosío Villegas”, Mexico City 14080, CP, Mexico; bsommer195@gmail.com; 5 Subdirección de Investigaciones Clínicas, Instituto Nacional de Psiquiatría Ramón de la Fuente Muñiz, Mexico City 14370, CP, Mexico; mvaldes_inprfm@yahoo.com; 6 Departamento de Neurobiología, Dirección de Investigación en Neurociencias, Instituto Nacional de Psiquiatría Ramón de la Fuente Muñiz, Mexico City 14370, CP, Mexico; ecalixto@imp.edu.mx; 7 Laboratorio de Biología Molecular, Departamento de Fibrosis Pulmonar, Instituto Nacional de Enfermedades Respiratorias “Ismael Cosío Villegas”, Mexico City 14080, CP, Mexico; araquiga@yahoo.com.mx; 8 Unidad de Epidemiología Hospitalaria e Infectología, Instituto Nacional de Enfermedades Respiratorias “Ismael Cosío Villegas”, Mexico City 14080, CP, Mexico; mcastillejos@gmail.com; 9 Departamento de Ciencias de la Salud, Universidad Autónoma Metropolitana-Iztapalapa, Mexico City 09340, CP, Mexico; hser@xanum.uam.mx; 10 Dirección de Investigación, Instituto Nacional de Geriatría, Mexico City 10200, CP, Mexico; jverjan@inger.gob.mx; 11 Laboratorio de Socioneurobiologia, Centro de Investigación en Ciencias Cognitivas, Universidad Autónoma del Estado de Morelos, Cuernavaca 62209, CP, Mexico; german.lopez@uaem.mx; 12 Laboratorio de Neurobiología Cognitiva, Centro de Investigación en Ciencias Cognitivas, Universidad Autónoma del Estado de Morelos, Cuernavaca 62209, CP, Mexico

**Keywords:** human olfactory neuronal stem cells, calcium signaling, PLCβ, IP_3_, schizophrenia

## Abstract

**Background**: Schizophrenia (SZ) is a multifactorial chronic psychiatric disorder with a worldwide prevalence of 1%. Altered expression of PLCβ occurs in SZ patients, suggesting alterations in the PLCβ/IP_3_/Ca^2+^ signaling pathway. This cascade regulates critical cellular processes in all cell types, including the neuronal lineage; however, there is scarce evidence regarding the functionality of this transduction signaling in neuronal cells derived from SZ patients. **Objective**: We evaluated the functionality of the PLCβ/IP_3_/Ca^2+^ pathway in olfactory neuronal precursor cells (hONPCs) obtained from SZ patients. **Methods**: Cryopreserved hONPCs isolated from SZ patients and healthy subjects (HS) were thawed. The cellular types in subcultures were corroborated by immunodetection of the multipotency and lineage markers SOX-2, Musashi-1, nestin, and β-III tubulin. The PLCβ/IP_3_/Ca^2+^ pathway was activated by GPCR (G_q_) ligands (ATP, UTP, serotonin, and epinephrine). In addition, PLCβ and IP_3_R were directly stimulated by perfusing cells with the activators m-3M3FBS and ADA, respectively. Cytosolic Ca^2+^ was measured by microfluorometry and by Ca^2+^ imaging. The amount and subcellular distribution of the PLCβ1 and PLCβ3 isoforms were evaluated by confocal immunofluorescence. IP_3_ concentration was measured by ELISA. **Results**: The results show that the increase of cytosolic Ca^2+^ triggered by GPCR ligands or directly through either PLCβ or IP_3_R activation was significantly lower in SZ-derived hONPCs, regarding HS-derived cells. Moreover, the relative amount of the PLCβ1 and PLCβ3 isoforms and IP_3_ production stimulated with m-3M3FBS were reduced in SZ-derived cells. **Conclusions**: Our results suggest an overall functional impairment in the PLCβ/IP_3_/Ca^2+^ signaling pathway in SZ-derived hONPCs.

## 1. Introduction

Schizophrenia (SZ) is a severe psychiatric disorder that usually presents its clinical onset in early adulthood and affects approximately 1% of the human population. The clinical symptoms that allow the diagnosis of this disorder are hallucinations, paranoia, inattention, decreased social interactions, lack of motivation, and cognitive impairment. SZ has a multifactorial etiology, and its development might have a complex genetic background but additionally involves diverse environmental risk factors [1,2]. Furthermore, SZ has been associated with altered neurotransmission mediated by serotonin, dopamine, glutamate, and GABA [3,4,5].

Diverse high-risk factors associated with SZ are encoded by genes related to calcium ion (Ca^2+^) signaling, suggesting that alterations in the pathways activated by this cation can be characteristic of this disorder [6]. Ca^2+^ is a key intracellular messenger in all cell types; it is versatile and regulates multiple subcellular processes involved in essential functions, such as proliferation, migration, differentiation, neurotransmission, and cell death, among others. In this regard, several studies have shown that the pathophysiology of SZ has been related to processes dependent on Ca^2+^ signaling, such as dysfunction in neuromodulation mediated by dopamine, glutamate, serotonin, and GABA, leading to malfunctioning of interneurons and consequently cognitive, behavioral, and social dysfunction [7]. Interestingly, several of these extracellular signals are transduced through activation of the PLCβ enzyme, which generates the second messenger IP_3_ and Ca^2+^ release from intracellular stores. In studies that analyze the expression of mRNA in biopsies of the orbitofrontal cortex, the deletion of the PLCβ1 gene in SZ patients has been suggested [8]; in addition, in post-mortem tissue, decreased levels of PLCβ1 mRNA expression in the dorsolateral prefrontal cortex [9], and lower levels of the PLCβ1 protein in the prefrontal cortex [10] were detected.

To the best of our knowledge, studies on the functionality of PLCβ in vivo in SZ patients are null. To overcome this limitation, models involving the isolation and culture of human cells from the neural lineage have been characterized, such as olfactory stem cells derived from the human olfactory epithelium (hOE) [11,12]. Experimental data suggest that isolated human olfactory neuronal precursor cells (hONPCs) that exhibit multipotent features can be propagated in culture and cryopreserved in biobanks. These hONPCs have been proposed to be a suitable model to study alterations found in neuropsychiatric and neurological diseases such as depression [13], Alzheimer’s disease [14,15,16], bipolar disorder [17], Parkinson’s disease [18], SZ [19,20,21], and in other pathologies such as fragile X syndrome [22], cannabis use [23], and COVID-19 and olfactory dysfunction [24].

The characteristics of hONPCs provide them with great potential as a human neural cell model, especially for studying neurodevelopmental disorders. The transcriptomic expression of these cells coincides with a mid-fetal stage of the brain. It has been used to describe the differential gene expression of multiple signaling pathway genes in SZ [20], which could serve for the further study and elucidation of the etiology of the disease and correlate embryonic and fetal events [20,25]. Furthermore, hONPCs have proven to be an effective model for studying cognition and neurodegenerative diseases, as Rantanen et al. observed while evaluating the transcriptomic profile of Alzheimer’s disease (AD) patients and patients with moderate cognitive impairment [15]. Furthermore, the unique qualities of hONPCs allow the potential identification of target genes and signaling pathways in diseased states, such as in SZ or AD, and the analysis of their possible role as therapeutic targets [21].

Due to the rigorous validation processes to which hONPCs have been subjected as a surrogate model, their robustness and reliability in providing consistent and accurate results in addressing pathophysiological mechanisms at the structural, cellular, and molecular levels have been guaranteed [12,26,27]. Therefore, the main objective of this study was to determine the functionality of the PLCβ/IP_3_ pathway through stimulation of PLCβ or by activating some G protein-coupled receptors (GPCRs) in hONPCs isolated from SZ patients and healthy subjects (HS).

## 2. Materials and Methods

### 2.1. Human Olfactory Neural Precursor Cells

This study was carried out in accordance with the Helsinki Declaration for human research; the donors of the olfactory epithelium samples previously signed an informed consent letter. This research was approved by the Institutional Bioethics Committee (Project number: INPRFM IC 092010.0). Highly trained specialists from the Schizophrenia Clinic of the Instituto Nacional de Psiquiatría Ramón de la Fuente Muñiz (INPRFM) performed clinical patient evaluations and diagnoses and referred the patients to this study. Thus, we used a convenience non-probabilistic sampling method, with both controls and patients being Mexicans and matched by age (±6 years). The patients had a range of 0–18 years from the onset of symptoms to the sample collection; two patients were untreated (naïve), and four patients were treated with standard antipsychotic drugs (haloperidol, fluoxetine, risperidone) by the time of the sample collection. There were no observable differences related to the demographic characteristics of the populations (Table 1).

Samples of the olfactory epithelium were obtained by exfoliation of the nasal cavity, as reported by Benítez-King and coworkers (2011), from subjects without psychiatric diagnosis (healthy subjects: HS) and patients diagnosed with SZ [12]. Briefly, cells were obtained with an interdental brush and mechanically dissociated in Dulbecco’s modified Eagle medium/F-12 nutrient mix (DMEM/F12), supplemented with 10% (*v*/*v*) fetal bovine serum, 2 mM L-glutamine, and 1% (*v*/*v*) penicillin–streptomycin. The dissociated cells were plated in a 4-well cell culture plate and incubated at 37 °C with 5% CO_2_ until the culture reached confluence. The cultures were replated in 25 cm^2^ cell culture flasks to obtain subcultures in different passages. These subcultures were cryopreserved in the supplemented DMEM/F12 medium with 8% DMSO. They were maintained submerged in liquid nitrogen in the cell bank of the Neuropharmacology Laboratory of the INPRFM. The experiments of this study were performed using cryopreserved subcultures of olfactory stem cells obtained from 6 HS and 6 SZ patients (Table 1); cells were thawed in passages 2 or 3, and experiments were carried out at passages 4–6.

### 2.2. Protein Detection by Immunofluorescence

Cells in passages 4–6 were placed on 12 mm diameter round coverslips and kept in culture with supplemented DMEM/F12 medium for three days with controlled temperature and CO_2_, 37 °C and 5%, respectively. Then, cells were fixed with 4% paraformaldehyde and permeabilized with 0.2% Tween-20 in PBS for 30 min; non-specific protein binding was blocked with 5% BSA for 1 h. Primary and secondary antibodies were titrated to determine their optimal concentrations for detecting their epitopes. Three marker proteins for multipotent stem cells were immunodetected using a commercial kit (R&D Systems^®^, Cat. NC025; Minneapolis, MN, USA): anti-SOX-2 (1:60), anti-Musashi-1 (1:60), and anti-nestin (1:60) were incubated overnight. In addition, the isoforms 1 and 3 of the enzyme PLCβ were immunodetected with a rabbit monoclonal antibody (1:10; Abcam Carlsbad, CA, USA, Cat. EPR18714). Both SOX-2 and Musashi-1 primary antibodies were detected by the DyLight™ 488-conjugated donkey anti-goat IgG (1:500; Invitrogen, Carlsbad, CA, USA, Cat. SA5-1086), whereas for detection of anti-nestin, cells were incubated with an Alexa Fluor™ 488-conjugated goat anti-rabbit IgG (1:500; Invitrogen, Cat. A32723). In the case of PLCβ 1 and 3, the secondary antibody was an Alexa Fluor™ 680-conjugated donkey anti-rabbit IgG (1:500; Invitrogen Cat. A10043). Cells were incubated for 60 min with the secondary antibodies at room temperature, and the nuclei were counterstained with 150 nM of 4′,6-diamidino-2-phenylindole (DAPI) for 4 min. Finally, coverslips were mounted with ProLong^TM^ Diamond Antifade Mountant (Thermo Fisher Scientific, Carlsbad, CA, USA, P36961). Labeling was observed on a ZEISS LSM 900 with a Airyscan 2 confocal microscope (Carl Zeiss Microscopy, Jena, Germany), and the images were analyzed by the ImageJ 1.53t and ZEN (blue edition, version 3.4.91) software. In all cases, non-specific fluorescence was assessed by omitting the primary antibodies, and, particularly for multipotent markers, J774A.1 mouse macrophages stimulated with LPS for 24 h were used as negative controls. Randomly chosen fields per subject were considered.

### 2.3. Quantification of Cytosolic Calcium Concentration by Microfluorometry

The functionality of various G protein-coupled receptors (particularly the Gq isoform) was examined by G protein activation in hONPCs from patients with SZ and HS. The cognate receptors present in these cells are described in Table S1 [23,28,29,30]. Cells in passages 3 to 5 at 80% confluence were detached with EDTA and a trypsin solution. Cells were seeded at 12,000 cells/cm^2^ density on rat tail collagen-coated round coverslips; the cultures were maintained under a controlled environment at 37 °C and 5% CO_2_ for three days with supplemented DMEM/F-12 medium.

Cells were incubated with 2.5 µM Fura 2-AM (Invitrogen), diluted in the supplemented DMEM/F-12 medium for 1 h at 37 °C and 5% CO_2_. Then, coverslips with cells were placed in a perfusion chamber on an inverted microscope (Diaphot 200, Nikon, Tokio, Japan) and perfused with Krebs solution at 37 °C with a 2–2.5 mL/min flow. Krebs solution contained (in mM): 118 NaCl, 25 NaHCO_3_, 4.6 KCl, 1.2 KH_2_PO_4_, 1.2 MgSO_4_, 11 glucose, and 2 CaCl_2_; pH was adjusted by aerating the solution with carbogen. The cytosolic Ca^2+^ concentration was quantified using a microphotometer (model D-104, Photon Technology International, Ford, West Sussex, UK), applying alternating light stimuli of 340 and 380 nm wavelengths and quantifying the 510 nm fluorescence emitted by Fura 2 bound to Ca^2+^. Light stimuli were applied at a frequency of 0.5 Hz, and intracellular Ca^2+^ concentration was calculated according to the Grynkiewicz formula [31]. The PLCβ/IP_3_/Ca^2+^ pathway was stimulated by perfusing cells with either 300 µM ATP, 300 µM UTP, 10 µM serotonin (5-HT), or 10 µM epinephrine (EPI). Additionally, Ca^2+^ measurements were performed by activating the PLC enzyme with a stimulus of 10 µM m-3M3FBS (non-specific activator, Tocris, Avonmouth, Bristol. UK Cat. 1941) [32]. Finally, the IP_3_R was stimulated directly with the specific activator adenophostin A hexasodium salt (32 nM ADA; Santa Cruz, Heidelberg, Germany, EU, Cat. sc-221213) [33]. Also, to evaluate GPCR ligand-induced Ca^2+^ responses not mediated through the PLCβ/IP_3_ pathway, hONPCs were perfused with either 10 µM dopamine (DOPA) or 10 µM glutamate (GLU). To compare the increase in intracellular Ca^2+^ concentration, we calculated the difference of the maximal amplitude minus the basal concentration in each cell. Data were obtained from 3 random responses from 6 subjects per group.

### 2.4. Calcium Imaging by Fluorescence Microscopy

Cells in passages 4–6 at 12,000 cells/cm^2^ density were cultured with supplemented DMEM/F-12 at 37 °C and 5% CO_2_ for three days. The Ca^2+^ indicator Fluo 4-AM (4 µM; Invitrogen) diluted in culture medium was added and incubated for 30 min at 37 °C and 5% CO_2_. Subsequently, cells were washed with Krebs solution at 37° C, and coverslips were placed in an epifluorescence microscope (Nikon Eclipse TE2000, Nikon, Tokyo, Japan). Intracellular Ca^2+^ increase was induced with either 300 µM ATP, 300 µM UTP, 10 µM 5-HT, 10 µM EPI, or 10 µM m-3M3FBS. Images were acquired with a Nikon digital camera (model DS-Ri2) and the NIS-Elements AR software (version 4.3); fluorescence was detected before stimulation (basal cytosolic Ca^2+^ concentration), and after 2 min (GPCR agonists) or 30 s (PLC activator), thapsigargin (1 µM, TG), and cyclopiazonic acid (10 µM, CPA) were added as the stimuli. The cells were manually segmented and the fluorescent marker intensity was quantified for each cell individually and represented as the mean fluorescence intensity in arbitrary units (MFI, AU, respectively). The images were analyzed using the software Fiji/ImageJ 1.54f. Data were obtained from 3 randomly selected fields from 6 subjects per group.

### 2.5. Measurement of IP_3_ Concentration by ELISA

Cells in passages 4–6 were plated in 75 cm^2^ culture flasks and cultured in supplemented DMEM/F12 medium. At 80% confluence, cells were stimulated with 10 µM m-3M3FBS for 20 min to activate PLC and increase the IP_3_ concentration. Cultures were washed with 4 mL pre-cooled PBS, and cells were detached with a trypsin-containing solution. Suspended cells were counted with a hemocytometer and centrifuged for 5 min at 1000× *g*. The supernatant was discarded, and cells were washed three times with pre-cooled PBS (200 µL of pre-cooled PBS was added for 1 × 10^6^ cells). Cells were frozen and thawed three times to be wholly lysed. The final centrifugation was performed at 4 °C and 1500× *g* for 10 min, and the supernatant was carefully collected.

The IP_3_ immunoassay was performed according to the ELISA kit manufacturer’s instructions (Abcam, Cat. ab287832). The optical density was measured at 450 nm, and measurements of IP_3_ for standards and samples were performed in duplicate and averaged. Finally, a four-parameter logistic curve was plotted in GraphPad Prism software (version 9.3.1) to obtain a standard curve and interpolate the values from HS and SZ cultures; data were normalized by pg/10^6^ cells and were obtained from 2 technical replicates of 4 subjects per group.

### 2.6. Statistical Analysis

Plotted data represent the mean ± standard error; data were compared using an unpaired Student’s *t*-test with Welch’s correction or a one-way analysis of variance (ANOVA). The significance of differences between groups was considered with *p* < 0.05. The sample size was determined using G*Power software (Version 3.1.9.6, Franz Faul, Universität Kiel, Germany) [34]. Using this software, we evaluated the effect of the population size using real data obtained in a pilot study. An a priori power analysis was performed, employing a two-tailed *t*-test or one-way ANOVA as applicable, with a significance level (α) set at 0.05. A power analysis (1-β) was selected at 80%. Statistical analysis was performed with GraphPad Prism software (version 9.3.1).

## 3. Results

### 3.1. Expression of Multipotency Markers in Cells from HS and SZ Patients

**Figure 1:**
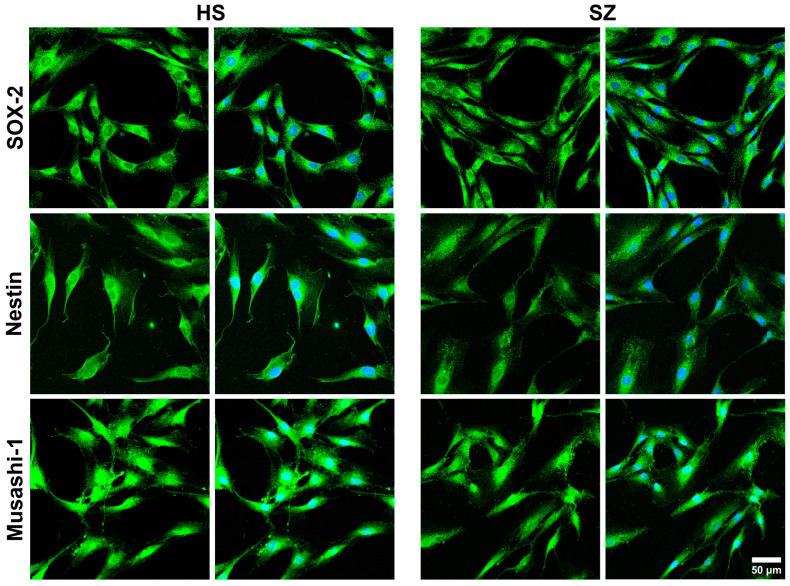
Determination of multipotency markers in hONPCs of HS and SZ patients. HS-derived and SZ patient-derived cells exhibit multipotent stem cell characteristics by expressing SOX-2, Musashi-1, and nestin. All cells express the three multipotency markers. DAPI-stained nuclei.

The present study found that hONPCs obtained from both HS and SZ exhibit characteristics of multipotent stem cells because they express molecular markers of multipotency, such as nestin, Musashi-1, and SOX-2 (Figure 1). Detecting these proteins suggests that cells from both patients and controls have equivalent characteristics of undifferentiated stem cells.

### 3.2. Olfactory Epithelium Single-Cell Ca^2+^ Response Induced by Gq-Coupled Agonists in HS and SZ Patients

**Figure 2:**
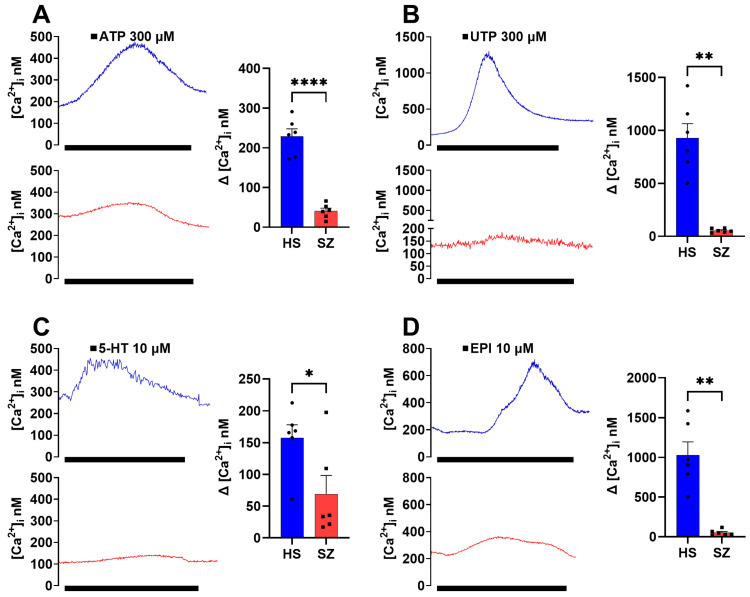
Increase in cytosolic Ca^2+^ induced by Gq-coupled agonists in single cells of the olfactory epithelium of HS and SZ patients. Cells were cultured for three days to assess changes in intracellular Ca^2+^ concentration ([Ca^2+^]_i_) induced by 300 μM ATP (**A**), 300 μM UTP (**B**), 10 μM serotonin (**C**), and 10 μM epinephrine (**D**) by microfluorometry using Fura 2-AM. The graphs represent the data obtained from six subjects per group. Each data point represents the average of three technical replicates for each subject. Data were expressed as mean ± SEM and compared using the Student’s *t*-test with Welch’s correction, * *p* < 0.05 ** *p* < 0.01 **** *p* < 0.001. HS = healthy subject (blue), SZ = schizophrenic patient (red).

In hONPCs, stimulation with GPCR (G_αq_) agonists (300 µM ATP, 300 µM UTP, 10 µM 5-HT or 10 µM EPI) induced a transient increase in the intracellular Ca^2+^ concentrations ([Ca^2+^]_i_) that returned to basal levels when the stimuli were removed (Figure 2). The Ca^2+^ responses induced by ATP, UTP, 5-HT, and EPI in HS-derived cells were higher than in SZ-derived cells. All groups were significantly diminished when comparing the ∆[Ca^2+^]_i_ of the response induced by the different GPCR agonists from the SZ patients’ cells regarding the HS-derived cells (Figure 2).

### 3.3. Cellular Population Ca^2+^ Imaging after GPCR Agonist Stimulation in hONPCs from HS and SZ Patients

**Figure 3:**
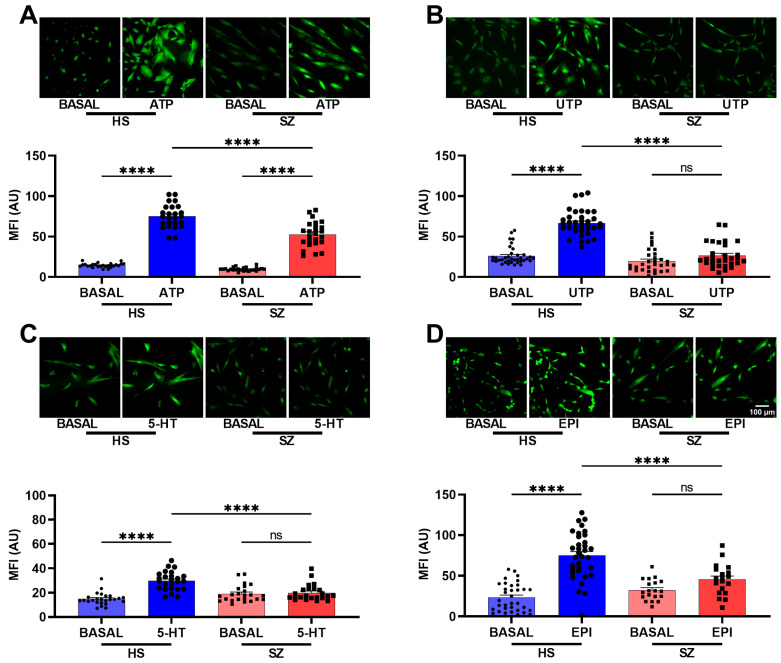
Intracellular calcium response after stimulation with G_q_-coupled agonists in olfactory epithelial cell populations of HS and patients with SZ. The cells were incubated for 30 min with 4 μM Fluo 4-AM diluted in the culture medium at 37 °C and 5% CO_2_. Images were captured before stimulation (basal) and 2 min after stimulation with either 300 μM ATP (**A**), 300 μM UTP (**B**), 10 μM serotonin (**C**), and 10 μM epinephrine (**D**) in HS-derived cells and SZ patient-derived cells. The graphs represent data obtained from at least 18 images (18–20 cells per image) per group. Data were expressed as mean ± SEM and compared using Brown–Forsythe one-way analysis of variance (ANOVA) and Dunnett’s multiple comparison test, **** *p* < 0.0001. ns: not significant. Mean fluorescence intensity (MFI), arbitrary units (AU).

**Figure 4:**
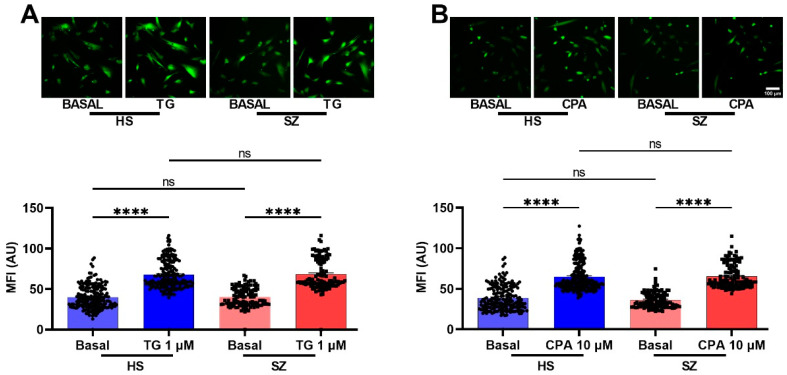
Intracellular calcium response after stimulation with thapsigargin and CPA in hONPCs of HS and patients with SZ. Images were captured before stimulation (basal) and 2 min after stimulation with either 1 μM thapsigargin (TG, left graph) (**A**), and 10 μM cyclopiazonic acid (CPA, right graph) (**B**) in HS and SZ derived cells. No significant differences were found when basal Ca^2+^ levels were compared (HS vs. SZ), neither when comparing treated cell responses. Highly significant differences were found when basal Ca^2+^ levels were compared with their respective treatment. The graphs represent data obtained from at least six images (18–20 cells per image) per group. Data were expressed as mean ± SEM and compared using the Kruskal–Wallis test and Dunn’s multiple comparisons test, **** *p* < 0.0001. ns: not significant. Mean fluorescence intensity (MFI), arbitrary units (AU).

**Figure 5:**
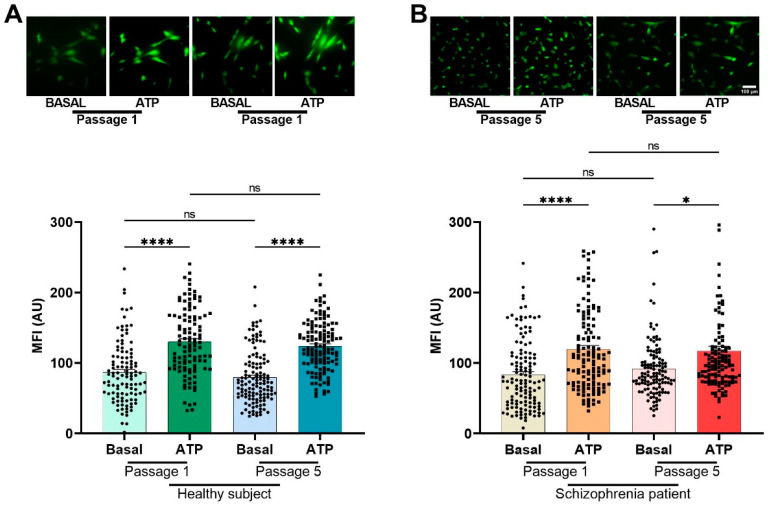
Intracellular calcium response after stimulation with ATP in olfactory epithelial precursor cell populations of HS (**A**) and SZ (**B**) patients in passage 1 and passage 5. The cells were incubated for 30 min with 4 μM Fluo 4-AM diluted in the culture medium at 37 °C and 5% CO_2_. Images were captured before stimulation (basal) and 2 min after stimulation with 300 μM ATP. Each data point represents the mean fluorescence intensity (MFI) measurement of each cell. The MFI of at least 100 cells was determined. The graphs represent data obtained from at least six images (18–20 cells per image) per group. Data were expressed as mean ± SEM and compared using the Kruskal–Wallis and Dunn’s multiple comparisons tests. **** *p* < 0.0001, * *p* < 0.05; not significant (ns). AU: arbitrary units.

To corroborate the differences in the Ca^2+^ responses observed in the microfluorometric studies, we performed Ca^2+^ imaging with Fluo-4 loaded hONPCs at the population level (Figure 3). To explore the non-receptor-mediated Ca^2+^ response from the internal stores, the cells were stimulated with thapsigargin (1 µM, TG) or cyclopiazonic acid (10 µM, CPA); we observed significant differences between basal Ca^2+^ levels and the corresponding stimuli, but not when compared between groups (HS vs. SZ) (Figure 4). The basal Ca^2+^ levels and the ATP-induced Ca^2+^ responses in passage 1 cells were not different from those observed in passage 5 of HS- and SZ patient-derived cells (Figure 5). The Ca^2+^ response in HS cells treated with either ATP, UTP, 5-HT, or EPI was higher when compared to the SZ cells and results suggest that differences in Ca^2+^ responses were not dependent on culture passage. These results suggest a generalized pattern of dysfunction in the Ca^2+^ response induced by GPCR (G_αq_) agonists, probably via an alteration in the PLCβ/IP_3_ pathway.

### 3.4. Function and Expression of the PLCβ Protein in hONPCs

**Figure 6:**
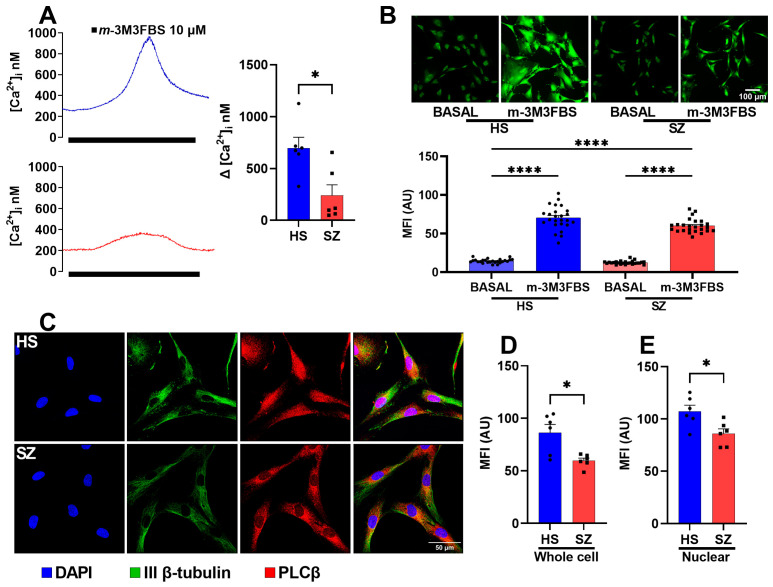
Cytosolic increase in Ca^2+^ after direct PLCβ stimulation and the amount of PLCβ protein in cultured cells from HS and patients with SZ. (**A**) Microfluorometric detection of changes in intracellular Ca^2+^ concentration ([Ca^2+^]_i_) induced by 10 µM m-3M3FBS. (**B**) Calcium images were captured before stimulation (basal) and 30 s after direct PLCβ stimulation (10 µM m-3M3FBS). (**C**) Immunofluorescent detection of PLCβ1 and 3; the panel shows representative images from cells detected by confocal microscopy. Neuronal-specific human IIIβ-tubulin (green) and PLCβ1 and 3 (red) were detected in the cells. Nuclei were counterstained with DAPI (blue). The mean fluorescence intensity of the whole cells (**D**) and of the nuclear zone (**E**) were compared. Data were expressed as mean ± SEM and compared using the Student’s *t*-test with Welch’s correction for panels (**A**,**D**,**E**) (* *p* < 0.05) or with one-way ANOVA and Tukey’s multiple comparisons test for panel (**B**) (**** *p* < 0.0001).

When a ligand (such as a neurotransmitter) binds to a GPCR (G_αq_) on the cell surface, it activates the G_αq_ protein subunit, subsequently activating PLCβ. Activated PLCβ cleaves phosphatidylinositol 4,5-bisphosphate (PIP2) into IP_3_ and diacylglycerol (DAG). IP_3_ then diffuses into the cytoplasm and binds to IP_3_ receptors (IP_3_R) on the endoplasmic reticulum, leading to the release of calcium ions from intracellular stores. To determine the functionality of PLCꞵ in the hONPCs, we stimulated isolated cells with 10 µM m-3M3FBS, an activator of PLCꞵ which is known to induce an intracellular Ca^2+^ increment [32]. In HS, the Ca^2+^ response was 696.2 ± 128.4 nM, n = 6, while in SZ patients this was, 239 ± 80.59 nM, n = 6, and we observed significant differences in the ∆[Ca^2+^]_i_ (*p* < 0.05) (Figure 6A). Similarly, we explored the overall Ca^2+^ response through Ca^2+^ imaging. There was no difference in the basal Ca^2+^ levels; however, the difference in the response to m-3M3FBS between HS (70.24 ± 3.01 AU) and SZ patients (59. 98 ± 1.778 AU) was significant (*p* < 0.001) (Figure 6B).

Since PLCβ1 and PLCβ3 are expressed in various tissues with differential subcellular distribution (PLCβ1 in the nucleus and plasma membrane; PLCβ3 is nuclear) and have a higher sensitization to G_αq_-mediated activation relative to other isoenzymes [35,36,37], we detected their amount and distribution in hONPCs from HS and SZ patients by confocal immunofluorescence (Figure 6C). In the cells of SZ patients, we found a significant decrease in the amount of PLCβ in the whole cell (SZ 57.58 ± 3.73 arbitrary units (AU) vs. HS 76.65 ± 7.261 AU) (Figure 6D). Interestingly, this pattern is also significantly lower at the subcellular level when comparing both groups at the nuclei (SZ 85.88 ± 5.7 AU vs. HS 107.2 ± 6.05 AU) (Figure 6E). These findings suggest that alterations in the expression of this protein may explain the decrease in [Ca^2+^]_i_ after a stimulus in the cells of SZ patients. Therefore, the altered Ca^2+^ response in the hONPCs of SZ patients could be independent of GPCR stimulation and directly caused by the lowered production of IP_3_ via PLCꞵ activation. This could contribute to altered signaling pathways and cellular processes associated with SZ.

### 3.5. Production of IP_3_ and IP_3_ Receptor Function in hONPCs

**Figure 7:**
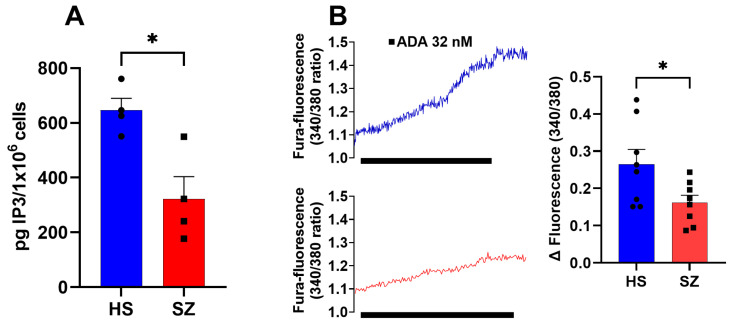
Effects of m-3M3FBS on the concentration of IP_3_ in hONPCs. (**A**) Cells of HS and SZ patients were stimulated with 10 µM m-3M3FBS for 20 min to activate PLCβ and increase the IP_3_ concentration. Data were normalized by pg IP_3_/10^6^ cells and obtained from two technical replicates of four subjects per group. (**B**) IP_3_R was directly stimulated with 32 nM ADA. The graphs represent data obtained from eight responses from six subjects per group. Data were expressed as mean ± SEM and compared using the Student’s *t*-test with Welch’s correction, * *p* < 0.05.

IP_3_ is produced through the action of PLCβ enzymes on PIP2, a phospholipid enriched at the cell membrane. In hONPCs (1 × 10^6^), a 20 min incubation with m-3M3FBS (a PLC activator) was used to stimulate the production of IP_3_; the intracellular concentration of inositol-triphosphate ([IP_3_]_c_) was determined by ELISA (Figure 7). In HS, the [IP_3_]_c_ was 646.1 ± 43.47 pg IP_3_/1 × 10^6^ (n = 4), while in SZ patients it was 322.5 ± 81.36 pg IP_3_/1 × 10^6^ (n = 4) (Figure 7A). We found significant differences among these groups in [IP_3_]_c_ production (*p* < 0.05). In addition, to determine the functionality of the IP_3_R, we stimulated hONPCs with ADA (a potent IP_3_R activator) to induce Ca^2+^ release from the sarcoplasmic reticulum [33]. In HS, the Δ fluorescence was 0.27 ± 0.04 (n = 8), and in the SZ patients, it was 0.16 ± 0.02 (n = 8) (Figure 7B). We found significant differences in the Δ fluorescence (340/380) (*p* < 0.05), showing a lower release of Ca^2+^ when the IP_3_R was activated in SZ cells.

### 3.6. Olfactory Epithelium Single Cell Ca^2+^ Response Independent of the IP_3_/IP_3_R/Ca^2+^ Pathway in HS and SZ Patients

**Figure 8:**
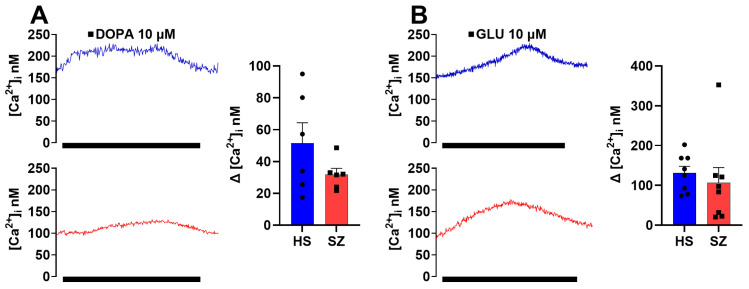
Increase in cytosolic Ca^2+^ induced by dopamine and glutamate in single cells of the olfactory epithelium of HS and SZ patients. Changes in intracellular Ca^2+^ concentration ([Ca^2+^]_i_) induced by 10 µM dopamine (**A**) and 10 µM glutamate (**B**) by microfluorometry using Fura 2-AM. The graphs represent data obtained from eight responses from six subjects per group. Data were expressed as mean ± SEM and compared using the Student’s *t*-test with Welch’s correction.

The Ca^2+^ responses in cells from HS induced by DOPA (51.54 ± 12.75 nM, n = 6) and GLU (131.1± 16.69 nM, n = 8) were similar to those observed for the SZ-cells: DOPA (31.80 ± 3.85 nM, n = 6) (Figure 8A), and GLU (106.8 ± 38.17 nM, n = 8) (Figure 8B), and no significant differences were observed when the Ca^2+^ responses were compared. This finding is understandable since the increase of cytosolic Ca^2+^ triggered upon ligand binding to the corresponding dopamine D_2_R and glutamate NMDA receptors is not mediated by IP_3_ production in hOE cells [30].

## 4. Discussion

hONPCs have different types of GPCRs with G_αq_ subunits. A cytosolic Ca^2+^ concentration increase was induced via the PLCꞵ/IP_3_ pathway upon ligand stimulation of these GPCRs with several ligands such as ATP, UTP, EPI, or 5-HT. These responses were attenuated in SZ patient-derived hONPCs, and, furthermore, activating PLCβ produced a similar diminished response. Since the production of IP_3_ and the function of IP_3_R were also reduced in the SZ-derived cells, it could be assumed that the alteration in the signaling pathway is directly related to the PLCꞵ activity, not due to alterations in the internal Ca^2+^ stores. The Ca^2+^ response induced by DOPA and GLU, which receptors do not signal through the PLCβ/IP_3_ pathway, consistently showed no difference between the HS and SZ groups. In this sense, a “convergent pathway hypothesis” can be suggested to emphasize the relevance of the PLCβ/IP_3_ pathway as a critical point of convergence of various dysfunctional neurochemical signals in SZ.

Previously, Durante et al. identified specific molecular markers in multipotent cells of the olfactory neuroepithelium, including horizontal basal cells (TP63, KRT5, CXCL-14, SOX-2, MEG3), globose basal cells (HES6, ASCL1, CXCR4, SOX-2, EZH2, NEUROD-1, NEUROG-1), respiratory horizontal basal cells (KRT5, TP63, SOX-2), and sustentacular cells (CYP2A13, CYP2J2, GPX6, ERMN, SOX-2) [38]. It has been noted that the expression of specific markers is a particularly significant advantage of using hONPCs. In previous works, markers in these cells, including nestin, Musashi-1, OCT3/4, NANOG, Notch, SOX-2, NCAM, and the neuronal IIIβ-tubulin, not only enabled the determination of their potency but also facilitated the verification of their proper isolation [28,39]. We should highlight that cells analyzed in this work were from early passages and had not been modified by a transforming virus or chemical compound. Furthermore, for the pathway we were interested in, the responses from cells at passage 5 or higher passages were not different than those found in earlier passages, as shown in Figure 5.

In SZ, reports of altered neurotransmitter-mediated signaling pathways, i.e., muscarinic, purinergic, glutamatergic, serotonergic, dopaminergic, and GABAergic, have been published [3,4,5]. The activation of these pathways initiates various cellular processes regulated by Ca^2+^, such as exocytosis, neuronal excitability, proliferation, differentiation, and neural plasticity [6,40,41]. SZ has been associated with Ca^2+^ signaling dysfunction, including the decreased activity of the NMDA receptor in early development stages [6]. There is evidence that the activity of NMDA receptors (NMDARs) is reduced when the phenotype of GABAergic inhibitory neurons is altered. There is also a decrease in the activity of serotonergic neurons located in the prefrontal cortex of patients with SZ [42,43]. Other proteins involved in Ca^2+^ signaling, such as the glutamate receptor mGluR5 [44] and muscarinic receptors [45], are also altered in SZ.

Interestingly, many extracellular signals involved in the pathogenesis of SZ are transduced through PLCβ-dependent pathways, implicating the function of this protein as a point of convergence in altered signaling. Four PLCβ isoenzymes (PLCβ1-4) encoded in different genes have been identified in mammals. These isoenzymes have differential distribution in tissues, e.g., PLCβ1 and PLCβ4 are found mainly in the brain, with exceptionally high expression in the cerebral cortex and hippocampus for PLCβ1 and in the cerebellum and retina for PLCβ4. On the other hand, PLCβ2 is preferentially present in hematopoietic cells, while PLCβ3 has ubiquitous expression. At the subcellular level, PLCβ1 is expressed on the plasma membrane, and all four PLCβ isoenzymes can be found in the nucleus. However, PLCβ1 appears to be the most abundant, followed by PLCβ3, PLCβ2, and finally PLCβ4 [36]. Dysregulation in the signaling associated with the different types of PLCβ is generally linked to various neuropsychiatric disorders, including epilepsy, Alzheimer’s disease, Huntington’s disease, bipolar disorder, depression, and SZ [8,46,47,48].

In this study, we observed an impaired function and a reduced amount of both the PLCꞵ1 and -ꞵ3 isoforms. PLCβ1 has been reported to be one of the first verifiable biomarkers that differentiate SZ from bipolar disorder [49]. In addition, studies performed on ex vivo samples from specific brain areas of SZ patients have reported alterations in PLCβ1, such as deletion of the PLCβ1 gene in the orbitofrontal cortex [46], decreased levels of PLCβ1 mRNA expression in the dorsolateral prefrontal cortex [9], and lower levels of the PLCβ1 protein in the prefrontal cortex [10]. In murine models, SZ-like behavior has been documented in phospholipase C β1 (PLCβ1(−/−)) knockout mice, such as hyperlocomotion, decreased exploration, nesting behavior, impaired working memory, and cognitive impairment, possibly due to abnormal cellular plasticity as a consequence of gene deletion and reduced mRNA and protein [50,51].

To the best of our knowledge, this is the first study providing evidence that the PLCβ signaling pathway is functionally impaired in cells from SZ patients, suggesting that previously reported abnormal levels of the enzyme may have a consequence on the functionality of its associated pathways, particularly in signal transduction through the PLCβ/IP_3_/Ca^2+^ cascade. This signaling pathway aims to generate and control highly complex Ca^2+^ signals, and the resulting increase in the concentration of cytosolic Ca^2+^ modulates various cellular functions, such as gene expression, metabolism, secretion, neuronal excitation, and cell death, among others [52,53]. Thus, a functional impairment at the level of PLCβ/IP_3_/Ca^2+^ might impact downstream elements of the signal transduction pathway, i.e., kinase activation, molecule translocation to specific subcellular compartments, cytoskeletal rearrangement, vesicle trafficking, etc.

We found a global change in the amount of PLCβ, and there are changes restricted to specific cellular compartments, such as the nucleus, where there is a reduced amount of PLCβ in SZ patients’ cells. Therefore, the measurement of PLCβ isoforms in different cell fractions can provide us with information on how this decrease in protein can affect specific molecular and cellular processes.

The heterogeneity of pathology and the poor efficacy of current classical therapeutic options that often either have an incomplete effect or hard-to-manage side effects has given rise to the search for new cell signaling pathways and drug target identification. Through GWAS and cellular response phenotype models, a myriad of genetic risk loci have been identified in PBMCs and iPSC-derived CNS cells, highlighting mechanistic points of convergence, such as epitopes of the Akt/GSK-3β pathway, the phosphorylation of CrkL, 4EBP1, and PLC-γ1, among others. Furthermore, the identification of a new compound, or repurposing of drugs, directed at the genetic risk loci, presents the possibility of personalized targeted therapeutic approach that could overcome the drawbacks of the current pharmacological options, such as treatment resistance. Some of the identified drugs include corticosteroids (methylprednisolone and flunisolide), potassium channel blockers (ibutilide), calcium channel blockers (nicardipine, nisoldipine), and thapsigargin (directed at PLC-γ1) [54]. Notably, the animal knockout models of PLC-γ1 that presents manic-like behavioral changes [55] and altered cell responses to thapsigargin has been associated with ATP2A2 mutations [56,57], indicating that PLC-γ1 could be a significant piece in the pathophysiology of SZ.

IP_3_, an essential cellular second messenger, is generated in response to the activation of specific receptors on the cell membrane. This messenger plays a fundamental role in the release of calcium within the cell [36]. Some studies show that SZ patients have altered levels of calcium in platelets. However, these findings are inconsistent, and a clear association has not been established. Arranz et al. [58] found that platelets’ IP_3_ concentrations at baseline and post-treatment with antipsychotics were not significantly different when compared to controls. Meanwhile, Rípová et al. [59] determined that [Ca^2+^]_i_ was significantly higher in platelets of neuroleptic-treated patients than in controls. Differences in IP_3_ levels were also found between controls and untreated and treated SZ patients. Studies have shown alterations in the levels and activity of enzymes involved in the synthesis and metabolism of IP_3_ in patients with SZ [46,60]. These alterations could contribute to altered neuronal function and symptoms associated with the disease. Importantly, SZ is a complex and multifactorial disorder, and the exact relationship between IP_3_ signaling dysfunction and SZ pathophysiology is not fully understood.

Our results provide new insights from an in vivo model that support the previous evidence obtained from the expression of PLCβ in postmortem brain tissues and clarify the divergent results that have been obtained up to now in the amount of IP_3_ in platelets of SZ patients, which are limited to be a model that simulates characteristics of bioaminergic neurons and catecholamine regulation [61,62]. Specifically, one of the most outstanding results in this work is the dysfunction in PLCβ activity, which leads to deficiencies in the production of the second messenger IP_3_ and the functionality of the IP_3_R, subsequently leading to an alteration in the release of calcium from intracellular stores in hONPCs. These alterations are relevant to increasing our understanding of SZ pathophysiology and could be a prospect for therapeutic targets and diagnostic tools. Nevertheless, further research is required to determine how this specific dysfunction is related to other neurochemical, genetic, and environmental factors involved in the development and progression of SZ.

The DOPA and GLU stimuli in hONPCs showed no difference between HS and SZ patients. The phenotypic receptor expression varies greatly, regulated by acute and chronic mechanisms to best fit its specific functions according to cell type, species, and stage of development to maintain a stable phenotype under physiological conditions and have the capacity to oversee the plasticity of the expression under new stimuli [63]. hOE cells have been reported to predominantly express the D_2_R isoform of the dopamine receptors, a G_i-_ and G_o-_coupled receptor. In contrast, for glutamate receptors, the predominant isoform in these cells is the NMDA receptor, an ionotropic receptor [30]. Both receptors are independent of the PLCꞵ signaling pathway and could explain the lack of difference between the responses observed in our study.

Some limitations of using hONPCs as a study model for SZ include the lack of information about the functionality of different signaling pathways in these cells. Additionally, due to the undifferentiated nature of these cells, it is necessary to confirm the results through conventional SZ models based on differentiated dopaminergic and serotoninergic neurons [64]. Although the statistical power of the present study enables the identification of significant differences between groups, our sample size is relatively small, and there is a need to increase the number of subjects using methodologies that allow us to work with a larger sample. We acknowledge that, up to this point, due to the heterogeneous nature of the pathology, no shared molecular mechanism found in all individuals diagnosed with schizophrenia is known.

hONPCs are a suitable model for studying cellular and molecular processes in neuropsychiatric disorders [26]. Furthermore, considering that these precursor cells are multipotent, the signaling impairment through the PLCꞵ/IP_3_/Ca^2+^ pathway may be conserved in their differentiated progeny, either neuronal or glial, implying that a broad spectrum of specialized functions could be altered. Thus, further research with hONPCs and their differentiated progeny would provide a deeper insight into how the altered PLCꞵ/IP_3_/Ca^2+^ pathway participates in diverse pathophysiological cellular processes in SZ.

## 5. Conclusions

The altered PLCβ/IP_3_/Ca^2+^ pathway in hONPCs may have broader implications that could contribute to dysfunctions underlying the pathophysiology of SZ. The dysregulation of the PLCβ/IP_3_/Ca^2+^ pathway and G_q_ ligand-triggered processes in these cells may impact their differentiation, migration, or survival, leading to structural and functional abnormalities in neuronal circuitries in SZ.

## Figures and Tables

**Table 1:** Sociodemographic data of sample donors. Olfactory neural precursor cells were obtained from 6 subjects per group. Cryopreserved cells were used in this study. HS corresponds to healthy subjects and SZ to patients diagnosed with schizophrenia.

Diagnosis	Sex	Age	Age at Diagnosis	Pre-Existing Conditions	Family Psychiatric History	Treatment	Time of Evolution	Alcoholism	Smoking
HS	F	28							
HS	F	23						Yes	
HS	F	27				Ibuprofen Vitamin C		Yes	
HS	M	23							
HS	M	27		Hypoglycemia				Yes	
HS	M	28		Hypothyroidism		Levothyroxine Coenzyme Q Carnitine			
SZ	F	32	25			Haloperidol 50 mg, biperiden 4 mg, fluoxetine 20 mg	7		
SZ	F	27	26			Risperidone 2 mg, fluoxetine 20 mg, biperiden 2 mg	1		
SZ	M	20	20	ADHD care at age 8		Naïve	0		
SZ	M	28	27			Naïve	1	Yes	Yes
SZ	M	31	28			Risperidone 2 mg	3		
SZ	F	33	15	Obesity and hypothyroidism	Yes	Haloperidol 10 mg, fluoxetine 40 mg, akineton 2 mg, eutirox	18		

## Data Availability

Data are available on request from the authors.

## Supplementary Materials

The following supporting information can be downloaded at: https://www.mdpi.com/article/10.3390/biomedicines12102343/s1, Table S1: G protein coupled receptors present in diverse tissues. Those recognized in ONPCs are designated by *. Methodology: Cryopreservation.
