# Supplementary material for: Altered PLCβ/IP3/Ca2+ Signaling Pathway Activated by GPRCs in Olfactory Neuronal Precursor Cells Derived from Patients Diagnosed with Schizophrenia
Source: Biomedicines. 2024 Oct 15;12(10):2343. doi: 10.3390/biomedicines12102343 (PMC11504003; doi:10.3390/biomedicines12102343)
Supplement: Supplementary file 1 [file biomedicines-12-02343-s001.zip › biomedicines-3233617-supplementary.pdf]

biomedicines

MDPI

Supplementary Materials

Methodology

Cryopreservation

1. Cell culture

Olfactory neural precursor cells (ONPCs) were cultured in Dulbecco's modified Eagle medium/nutrient mixture F-12 (DMEM/F12), supplemented with 10% (v/v) fetal bovine serum, 2 mM L-glutamine, and 1% (v/v) streptomycin-penicillin at 37°C with 5% CO_2, until 80% confluence was reached.

2. Cell trypsinization

Once the culture reached confluence, the culture medium was aspirated and the cells were washed with PBS. The cells were then detached using a 0.25% (w/v) trypsin-EDTA solution (Gibco). After 2 minutes, the enzymatic activity of trypsin was inactivated with supplemented medium and the cells were centrifuged at 1000g for 5 minutes.

3. Cell Suspension

The supernatant was discarded and cells were carefully resuspended in cryopreservation medium (8% (v/v) DMSO in supplemented culture medium). $1x10^6$ cells/ml were dispensed into sterile 1.8 ml cryovials (Corning).

4. Controlled Freezing/Long-Term Storage

Cryovials were placed in an ultra-freezer at -70°C for 24 hours, where cells were gradually frozen at a rate of 1°C/minute, using a controlled freezing container (Mr. Frosty, Nalgene). Subcultures were then stored in liquid nitrogen for long-term cryopreservation.

Table S1. G protein coupled receptors present in diverse tissues. Those recognized in ONPCs are designated by *.

Agonist	PGαq (PLCβ/IP3/Ca^{2+})	PGαs (AMPc)	PGαi/o (AMPc)	References
ATP	P2Y1, 2*, 4*, 6*, 11*	P2Y11*	P2Y12, 13, 14	[23,28]
UTP	P2Y2*, 4*			[23,28]
Serotonin	5-$HT2_{A*, B, C*}$	5-$HT1_{A, B, D-F}$<br>5-$HT3_A$<br>5-$HT4_{A-H}$	5-HT_{5A}<br>5-HT_6<br>5-$HT7_{A-D}$	[23,29,28]
Epinephrine	α1, β1, β2, β3		α2, β2, β3	[28]
Dopamine		D1, D5	D2*, D3, D4	[28,29]
Glutamate	mGluR1, 5	mGluR1, 5	mGluR2, 3, 4, 6, 7, 8	[28]
